# Spontaneous visual exploration during locomotion in patients with phobic postural vertigo

**DOI:** 10.1007/s00415-020-10151-8

**Published:** 2020-08-27

**Authors:** J. Penkava, S. Bardins, T. Brandt, M. Wuehr, D. Huppert

**Affiliations:** 1 German Center for Vertigo and Balance Disorders (DSGZ), Ludwig-Maximilians-University, Marchioninistr. 15, 81377 Munich, Germany; 2 Institute for Clinical Neurosciences, Ludwig-Maximilians-University, Munich, Germany; 3 Department of Neurology, University Hospital, Ludwig-Maximilians-University, Munich, Germany

**Keywords:** Phobic postural vertigo, Functional dizziness, Visual exploration, Eye movements, Head movements

## Abstract

**Background:**

Earlier studies on stance and gait with posturographic and EMG-recordings and automatic gait analysis in patients with phobic postural vertigo (PPV) or visual height intolerance (vHI) revealed similar patterns of body stiffening with muscle co-contraction and a slow, cautious gait. Visual exploration in vHI patients was characterized by a freezing of gaze-in-space when standing and reduced horizontal eye and head movements during locomotion.

**Objective:**

Based on the findings in vHI patients, the current study was performed with a focus on visual control of locomotion in patients with PPV while walking along a crowded hospital hallway.

**Methods:**

Twelve patients with PPV and eleven controls were recruited. Participants wore a mobile infrared video eye-tracking system that continuously measured eye-in-head movements in the horizontal and vertical planes and head orientation and motion in the yaw, pitch, and roll planes. Visual exploration behavior of participants was recorded at the individually preferred speed for a total walking distance of 200 m. Gaze-in-space directions were determined by combining eye-in-head and head-in-space orientation. Walking speeds were calculated based on the trial duration and the total distance traversed. Participants were asked to rate their feelings of discomfort during the walk on a 4-point numeric rating scale. The examiners rated the crowdedness of the hospital hallway on a 4-point numeric rating scale.

**Results:**

The major results of visual exploration behavior in patients with PPV in comparison to healthy controls were: eye and head positions were directed more downward in the vertical plane towards the ground ahead with increased frequency of large amplitude vertical orientation movements towards the destination, the end of the ground straight ahead. The self-adjusted speed of locomotion was significantly lower in PPV. Particularly those patients that reported high levels of discomfort exhibited a specific visual exploration of their horizontal surroundings. The durations of fixating targets in the visual surroundings were significantly shorter as compared to controls.

**Conclusion:**

Gaze control of locomotion in patients with PPV is characterized by a preferred deviation of gaze more downward and by horizontal explorations for suitable auxiliary means for potential postural support in order to prevent impending falls. These eye movements have shorter durations of fixation as compared to healthy controls and patients with vHI. Finally, the pathological alterations in eye–head coordination during locomotion correlate with a higher level of discomfort and anxiety about falling.

## Introduction

Phobic postural vertigo (PPV), a subtype of persistent postural-perceptual dizziness (PPPD), is a chronic functional disorder characterized by dizziness and subjective imbalance during stance and gait despite normal performance in vestibular and clinical balance tests [1–3]. This condition is the second most common diagnosis in an interdisciplinary tertiary outpatient dizziness unit (German Center for Vertigo and Balance Disorders) [4]. Neurophysiological analysis of stance and gait in PPV revealed an inadequate musculoskeletal stiffening with co-contraction of anti-gravity muscles [5–9]. The impairment of gait correlates with subjective fear of falling and balance confidence, although patients with PPV do not exhibit a higher rate of falls compared to healthy controls [10]. The stiffened postural control by muscle co-contraction is typical for PPV, but not specific, since stance and gait in individuals with visual height intolerance (vHI) or acrophobia exhibit similar stiffening of anti-gravity muscles when exposed to heights [11, 12]. Walking of patients with PPV and of those susceptible to vHI is similarly characterized by a reduced speed, shorter steps with decreased cadence, and longer times in double support [8, 13]. Ocular motor behavior during stance and locomotion has also been tested in individuals with vHI when exposed to heights by use of a mobile infrared eye-tracking system with integrated inertial sensors for monitoring of head movements [14–16]. Visual exploration of the surroundings and the abyss during stance [14] and during walking at heights [15] when measured as ‘gaze-in-space’ behavior was significantly restricted in susceptible subjects whereas non-susceptible subjects freely explored the entire visual field and the abyss.

The current study is focused on visual exploration of patients with PPV during locomotion. This is of relevance for the pathophysiological interpretation of the motor behavior in both disorders. In vHI, the combined pattern of reduced mobility of legs, neck, and eyes was interpreted as an atavistic motor reaction (primitive reflex) to the phobic stimulus of height [16]. The question for the current investigation is whether the typical impairments of stance and gait control are also accompanied by a restriction of eye and head movements in patients with PPV.

## Methods

### Subjects

Twelve patients with PPV (5 females, age: 28–86 years, mean 57.2 years) and eleven healthy controls without any psychiatric, neurologic, vestibular, or balance disorders (6 females, age: 23–52 years, mean 30.0 years) participated in the study. The diagnosis of PPV was based on the established diagnostic criteria [1, 17, 18]. Each patient underwent a detailed diagnostic work-up including thorough history-taking, clinical-neurological examination, functional-vestibular testing including orthoptic examination, caloric irrigation, and video head-impulse test.

### Experimental setup and procedures

**Fig. 1:**
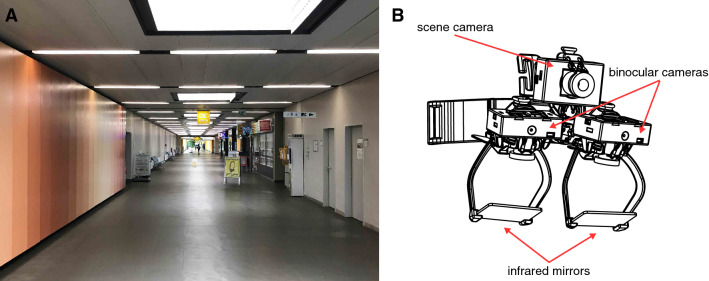
**a** Participants walked at a self-paced speed on a hospital hallway for a total distance of 200 m separated by 4 recording blocks with intermittent breaks. **b** Visual exploration behavior (i.e., head and eye-in-head orientation and motion) was recorded by a mobile infrared video eye-tracking system, consisting of goggles, a head-fixed camera, a head-fixed inertial measurement unit, and a backpack with a recording laptop

The main walkway of the University Clinic (about 450 m long) with shops and cafés on one side (Fig. 1a) was used as the experimental site for studying visual exploration of patients in crowded situations. Prior to the experiment, participants were equipped with a mobile infrared video eye-tracking system consisting of goggles, a head-fixed camera, and a backpack with a recording laptop (EyeSeeTec GmbH, Munich, Germany, sampling rate of 220 Hz) that measured eye movements in the horizontal and vertical planes (Fig. 1b). Eye movements were calibrated with a 5-point protocol. The calibration dots were projected from a laser unit attached to the head-fixed camera. Head orientation and motion in the yaw, pitch, and roll planes were measured with an inertial measurement unit (IMU) containing a triaxial accelerometer, gyroscope, and magnetometer (APDM, Inc., Portland, OR, sampling rate of 128 Hz). The eye tracker and inertial measurement unit recordings were synchronized by an external analog trigger signal (NI USB 6008, National Instruments).

During the experiment, participants were asked to walk at a self-paced speed for a given distance without any advice on how to visually explore their surroundings. The visual exploration behavior of participants was subsequently recorded four times, for a total walking distance of 200 m. The time required in seconds to cover this distance was measured. Repeated trials were performed to examine potential habituation effects. After accomplishing the four recordings, participants were asked to rate their feelings of discomfort on a 4-point numeric rating scale (0 = no, 1 = minor, 2 = medium, 3 = intense). The crowdedness of the hallway during recordings was estimated by the examiner on a 4-point numeric rating scale (0 = empty, 1 = little, 2 = moderately, 3 = highly crowded).

### Data analysis

Data were stored for off-line analysis and analyzed with MATLAB (The Mathworks Inc. Version 2019b). Head orientation (with respect to the central perspective vanishing point of the walkway) and head angular velocity in the horizontal (yaw) and vertical (pitch) planes were calculated based on the IMU recordings. Head orientation estimates derived from IMU recordings were calibrated at the beginning of recordings based on manually identified fixed points in the scene camera recording. Eye movement data (from the left eye) was processed to identify fast phases (saccades) and slow phases (fixations, smooth pursuit, vestibulo–ocular reflex), respectively. For this purpose, eye velocity was calculated by 3-point differentiation and subsequent Gaussian low-pass filtering using a cut-off frequency of 30 Hz. Fast phases were detected automatically using a threshold of 100°/s of maximal (peak) eye velocity. The onset and offset of each saccade were defined by a threshold of 5% of saccade peak velocity. Recording periods that contained eye blink artifacts were excluded from the analysis. The horizontal and vertical position of each saccade endpoint was calculated with respect to the central perspective vanishing point of the walkway. Durations of fixation were calculated as the intervals between separate fast phases of eye movements. Gaze-in-space directions were determined by combining head orientation and eye-in-head orientations. Walking speeds of patients and controls were calculated based on the trial duration and the total distance covered.

### Statistical analysis

Descriptive statistics are reported as mean ± SD. Differences of visual exploration behavior between patients and healthy controls were assessed by a linear mixed model with the factors group (healthy vs. patients) and trial on the following outcome measures: walking speed, subjective discomfort and walkway crowdedness, mean and SD of head orientation, mean angular head velocity, mean and SD of eye orientation, and fixation duration. The relationship between the visual exploration characteristics in patients and both the individual degrees of discomfort and the level of walkway crowdedness was analyzed by Spearman’s rank correlation. Results were considered significant at *p* < 0.05. Statistical analysis was performed using SPSS (Version 25.0; IBM Corp., Armonk, NY).

## Results

**Fig. 2:**
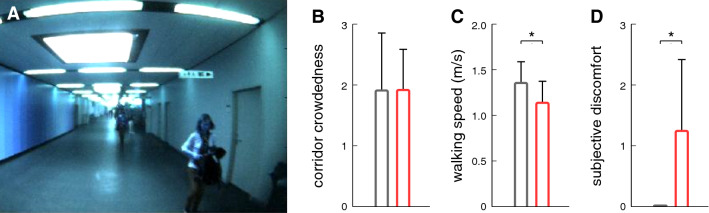
**a** Exemplary view of the visual surroundings during an experimental trial. Comparison of **b** the level of crowdedness of the corridor during individual trials, **c** walking speed, and **d** levels of discomfort between patients with PPV (red bars) and healthy controls (gray bars) on a 4-point numeric rating scale. Despite equally crowded scenery on average across individual recordings, patients walked considerably slower and frequently reported feelings of discomfort, imbalance, or dizziness

**Fig. 3:**
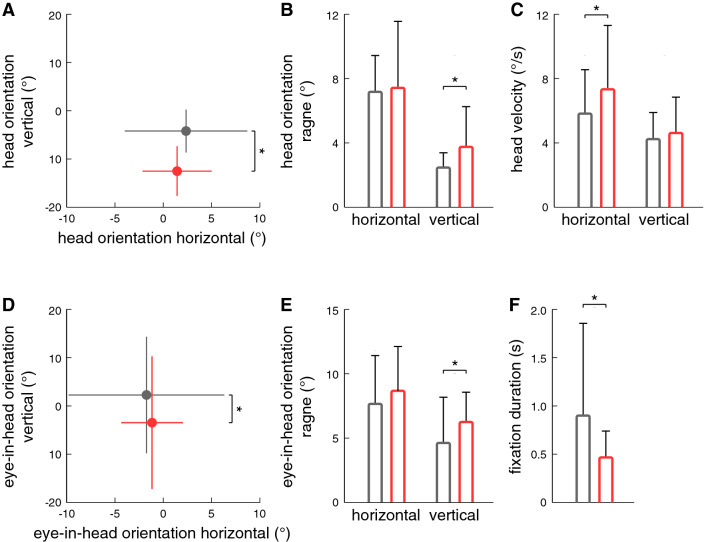
Comparison of **a** mean and **b** range of head orientations, **c** mean head velocity, **d** mean and **e** range of eye-in-head orientations, and **f** fixation duration during walking between patients with PPV (red dots, bars) and healthy controls (gray dots, bars). During walking, eye and head positions of patients were directed more downward in the vertical plane towards the ground ahead with increased frequency of large amplitude vertical orientation movements towards the destination, the end of the floor straight ahead and an overall shorter fixation duration

**Fig. 4:**
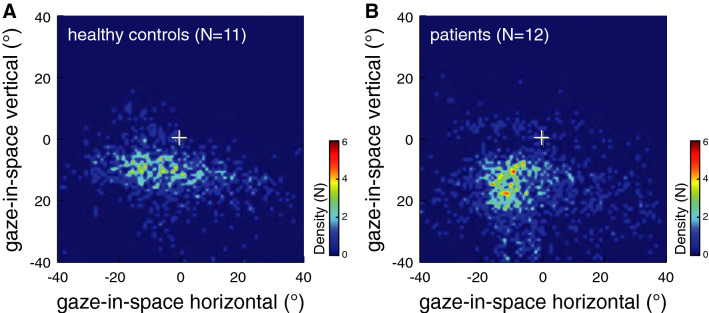
Comparison of gaze-in-space behavior in **a** healthy controls and patients with PPV (**b**). Density plots represent fixations of environmental structures with combined head and eye-in-head movements during walking. The number of participants (N; coded by color) fixating identical targets (resolution of 1° horizontally and vertically) centered around the central perspective vanishing point of the walkway (white cross) are depicted. Patients directed their gaze more along the vertical plane with a preference for downward orientations towards the ground ahead

The amount of people present in the experimental environment varied slightly between different recording days; however, the average level of crowdedness did not differ between recordings in patients and healthy controls (Fig. 2b). While healthy participants did not experience any discomfort during the experiment, patients walked at a slower pace (*p* = 0.002; Fig. 2c) and frequently reported feelings of discomfort while walking along the walkway (*p* < 0.001; Fig. 2d). Subjective imbalance and discomfort in patients were linked to objectively measurable alterations in their visual exploration behavior. Patients’ average head and eye-in-head orientation was directed more downwards (*p* < 0.001; Fig. 3). The tendency to direct gaze-in-space more downwards (Fig. 4) was accompanied by an increased range of vertical head and eye movements (*p* < 0.001), which enables the patients to visually screen the straight-ahead direction from time to time. Patients further exhibited increased angular head velocities particularly in the horizontal plane (*p* = 0.041).

The average duration of visual fixations was considerably reduced in patients compared to healthy controls (*p* = 0.005; Fig. 3f). Alerted visual exploration behavior did not show any habituation effects during the course of the four repeated walks.

**Fig. 5:**
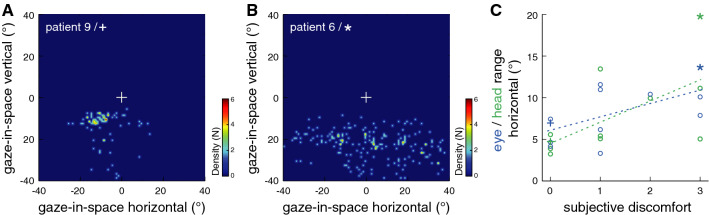
Exemplary gaze-in-space behavior in **a** a patient with PPV reporting no discomfort (patient 9/**+**) compared to **b** a patient with PPV reporting intense discomfort (patient 6/*) during the experiment. Density plots represent fixations of environmental structures with combined head and eye-in-head movements during walking. The number of fixations (N; coded by color) focusing on identical targets (resolution of 1° horizontally and vertically) centered around the central perspective vanishing point of the walkway (white cross) are depicted. **c** Correlation between individual levels of subjective discomfort on a 4-point numeric rating scale and the range of horizontal eye and head orientations in patients with PPV. Particularly anxious patients exhibited a more or less pronounced nervous visual exploration of their horizontal surroundings to a degree that depended on their level of subjectively reported discomfort

Between individual patients, both the level of subjective discomfort and the level of walkway crowdedness determined further characteristic alterations, in particular with respect to a more vivid visual exploration in the horizontal plane (Fig. 5). Accordingly, patients reporting a higher level of discomfort exhibited an increased range (*R*^2^ = 0.61; *p* = 0.035) and velocity (*R*^2^ = 0.61; *p* = 0.037) of horizontal head movements as well as an increased horizontal range of eye-in-head orientations (*R*^2^ = 0.59; *p* = 0.045). Analogously, a higher level of walkway crowdedness during experiments was associated with increased horizontal head velocities (*R*^2^ = 0.62; *p* = 0.031).

## Discussion

In PPV patients, the analysis of visual exploration by eye and head movements during straight ahead locomotion in a crowded hospital hallway revealed significant differences to healthy controls as well as to previous reports on visual exploration behavior in subjects susceptible to vHI. Major differences to healthy controls were: eye and head positions were directed more downward in the vertical plane towards the ground ahead with increased frequency of large amplitude vertical orientation movements towards the end of the ground straight ahead. Particularly patients that reported high levels of discomfort exhibited a more or less pronounced visual exploration of their horizontal surroundings with staccato-like eye-movements, which can be interpreted as a nervous visual exploration. Major differences of visual exploration compared to subjects susceptible to vHI were: eye and head movements were less restricted in PPV for vertical and horizontal planes and durations of fixation times were significantly shorter. In the following, we will first shortly summarize earlier findings of stance and gait parameters in both conditions and then discuss the characteristics and differences of visual exploration behavior.

### Stance and gait in PPV and vHI

In patients with PPV, posturographic and EMG measurements of muscular activity during free upright stance revealed a pathological increase of body sway activity and an inadequate co-contraction of anti-gravity leg and neck muscles, resulting in a stiffening of body posture. This continuous anxious control of posture which is linked to muscle co-contractions [5, 6, 11] leads to a kind of vicious circle [7, 9, 16]. Distraction of the patients by mental dual-tasking normalizes leg muscle activity and balance [9]. In healthy subjects and in subjects susceptible to vHI, exposure to heights causes similar alterations of postural control. It has been shown by Carpenter and colleagues in healthy subjects that increased postural threat by standing on elevated surfaces in the laboratory causes musculoskeletal stiffening of postural control [19] associated with changes in vestibulo–spinal reflexes [20]. The stiffening of anti-gravity muscles could also be found in subjects susceptible to vHI when exposed to real or virtual heights on an open escape balcony [11, 12]. Thus, the above described motor pattern is typical, but not specific, since it holds for both anxiety-related conditions, PPV and vHI.

The same is true for the characteristic gait patterns in both conditions. The gait of patients with PPV is characterized by slow speed, reduced cadence and stride length, and increased double support [8], a pattern earlier described for gait changes in healthy subjects exposed to an increased postural threat [21–23]. The gait at heights of subjects susceptible to vHI and acrophobia showed similar features as that found in patients with PPV. It is reminiscent of a strategy of cautious gait control that healthy persons adopt to avoid falls when walking on ice. Anxiety appears to be the critical symptom that causes the typical motor behavior, since improvements could be observed by cognitive dual-tasking at heights which reduces the actual anxiety level [13].

### Visual exploration behavior during locomotion in PPV compared to vHI

Vision has a major role within the multisensory balance control during stance [24, 25] and gait [26]. During locomotion, a coordination is necessary between the biomechanics of the bipedal gait cycle and gaze-in-space that gathers the spatial information required for the maintenance of the direction of movement and to determine safe locations for foot placements [27]. The previous literature on the role of visual gait control about upcoming footholds emphasizes regular ground fixations at two step lengths ahead [28–30]. The constant time of looking ahead was determined to be 1.5 s for avoidance of hazardous obstacles independent of the terrain conditions [31, 32]. Gait analyses by full-body kinematics combined with simultaneous recording of eye tracking revealed that on flat terrain the role of vision is modest with ground fixations occurring only during about half of the walking time [27]. This is in agreement with earlier findings by Pelz and Rothkopf [33]. The results of visual exploration in patients with PPV in a hallway in the hospital with a smooth surface without obstacles showed inadequate gait control with eye-in-head orientation mainly directed more downwards as compared to healthy controls, whose gaze was directed relatively higher with respect to the surroundings. This may be related to the increased fear of falling in PPV patients who adopt an inappropriate strategy which healthy persons only use while walking on unsafe terrain. Large amplitudes of vertical gaze-in-space movements reflect the continuous changes between the two fixations of the ground two steps ahead and the destination of locomotion straight ahead. Patients appear to feel the need to visually screen the straight-ahead direction from time to time. This vertical exploration of the surroundings had also been observed in subjects susceptible to vHI during locomotion at heights [15]. In both conditions, the preferred vertical exploration of the surroundings is anxiously driven to avoid either “falling down” on the ground in PPV or “falling off” a cliff in vHI [16].

In PPV patients, visual exploration in the horizontal plane was characterized by nervous, staccato-like eye and head movements scanning the surroundings. This was especially observed in those patients that reported high levels of discomfort presumably reflecting their anxiety. This is in contrast to persons with vHI, in whom the explored area was restricted towards the wall of the building and the handrail, but who simultaneously avoided looking towards the open side of the balcony [15]. Durations of target fixations were different in both conditions: they were considerably reduced in patients with PPV compared to healthy controls, whereas in vHI there was a tendency to longer durations of fixations [15]. This eye–head orientation pattern in PPV patients is best explained by an anxious search for suitable auxiliary means for potential postural support. Analogously, a similar kind of nervous horizontal visual search has been found in other neurological disorders, in which ocular motor control is driven by anxiety, such as in patients with cognitive disturbances of spatial orientation in navigational tasks. Patients with amnestic mild cognitive impairment, for instance, showed an increased frequency of horizontal eye movements and fixations, obviously to regain orientation by searching for visual landmarks to find the correct walkway to the envisaged goal [34, 35]. This horizontal visual search was reflected by activations of the pontine ocular motor center for horizontal eye movements in FDG-PET immediately following the navigational tasks [34]. Patients with chronic bilateral vestibulopathy, which also impairs spatial memory and navigation [36–39], showed a higher frequency of horizontal fixation saccades, particularly at junctions or crossings of walkways [40]. With respect to acrophobia, which is psychiatrically defined as a specific phobia [41, 42], the exploratory behavior of patients with specific phobias such as animal or social phobias must be mentioned [16]. The general visual exploration pattern in patients with specific phobias when exposed to the threatening stimulus is that of a “hypervigilance-avoidance” exploration. Subjects with spider phobias, for example, detect the critical spiders faster in the initial presentation phase, but subsequently shift their view more often away from the spiders as compared to controls [43–45]. The same was described for subjects with an injection phobia [46] or social phobias [47]. The latter form of avoidance behavior may present an alternative explanation for the pattern of eye and head movements in PPV-patients in the sense of avoiding looks from oncoming individuals. One important limitation of the study might bias the present findings: due to the different age distributions between patients and controls, we cannot exclude the possibility that differences in patients’ visual exploration behavior may be partly explained by age-related effects.

In conclusion, gaze control of locomotion in patients with PPV is characterized by a preferred deviation of gaze more downward towards the ground and by nervous horizontal explorations in search of possible support to avoid impending falls. These eye movements have shorter durations of fixations as compared to healthy controls and patients with vHI. The pathological alterations in eye–head coordination during locomotion correlate with a higher level of discomfort and anxiety about falling. The exploration pattern in PPV patients is mainly different to that of patients with vHI in two aspects: amplitude and frequency of eye–head movements are increased in PVV with shorter duration of fixations of visual exploration. However, postural control of stance and gait are very similar in both conditions.
